# Blended learning and student engagement: a case of undergraduate business students in Nepal

**DOI:** 10.3389/fpsyg.2026.1805135

**Published:** 2026-03-27

**Authors:** Som Nepali

**Affiliations:** Divya Jyoti Community Organization, Hetauda, Nepal

**Keywords:** active learning, blended learning, business education, collaborative learning, digital literacy, mixed-methods research, Nepal, student engagement

## Abstract

Blended learning is increasingly adopted in higher education, yet its impact on student engagement in Nepal remains underexplored. This study examined how undergraduate business students experience blended learning and its effects on knowledge, engagement, and collaboration. Using a sequential mixed-methods design, 120 students completed pre- and post-tests and structured engagement surveys, while 12 students participated in focus group interviews. Quantitative data were analyzed with descriptive statistics and the Wilcoxon signed-rank test, and qualitative data were examined through thematic analysis. Findings revealed that students actively engaged in discussion forums, case-based problem solving, group projects, and gamified quizzes, which enhanced critical thinking, teamwork, and comprehension of business concepts. Post-test results indicated significant improvements in knowledge and engagement (*Z* = −5.127, *p* < 0.001). Students reported greater motivation, interest, and confidence in learning, highlighting the role of digital tools in supporting flexibility and collaboration. Challenges included unreliable internet, unfamiliarity with digital platforms, and balancing online and in-person tasks. The study concludes that structured blended learning, supported by active learning strategies and digital literacy, enhances student engagement and skill development. Future research should explore other disciplines, longer-term effects, teacher perspectives, and the interplay of digital access and student-centered pedagogy to inform effective implementation in Nepalese higher education.

## 1 Introduction

Since the early 2000s, researchers in higher education have studied topics like technology in learning, student-centered teaching, learning outcomes and classroom participation (Alam et al., 2023; Bastola et al., 2024). More recently, studies have shown that even though blended learning is becoming popular worldwide, there are still very few studies on it in developing countries (Abednego et al., 2023). This is especially true in South Asia, where digital technology and teaching practices differ a lot between institutions (Jailani et al., 2025; Topping et al., 2022). While research in developed countries has looked at how blended learning affects student performance, satisfaction and participation (Dorji, 2024; Leonardo, 2025), there is still limited knowledge about how it helps engage undergraduate students in Nepal.

The Ministry of Education, Science and Technology in Nepal has encouraged the use of digital tools and student-centered teaching to improve higher education (Divjak et al., 2022). Following this policy, universities and colleges have started using blended learning more, especially after the COVID-19 pandemic, which increased the use of online teaching platforms (Ahmed et al., 2022b; Kadel et al., 2025). Blended learning, which mixes in-person teaching with online tools, is seen as a good way to make learning more flexible, accessible and engaging for students (Bidari et al., 2023; Khanal, 2024). However, it is still unclear how well these methods help students participate and stay engaged, particularly in undergraduate business programs where skills like critical thinking, teamwork and problem solving are very important.

Student engagement has been widely discussed in educational psychology and is often considered a key factor influencing learning outcomes. One of the most widely cited frameworks is the multidimensional model of engagement proposed by Burton and Yang (2025), which conceptualizes engagement as consisting of three interconnected components: behavioral engagement, emotional engagement, and cognitive engagement. Behavioral engagement refers to students’ participation in academic activities such as discussions, assignments, and collaborative tasks. Emotional engagement reflects students’ interest, motivation, and positive attitudes toward learning activities (Yan et al., 2025). Cognitive engagement relates to students’ effort to understand complex ideas, think critically, and apply knowledge in meaningful ways (Pham Thi et al., 2025; Wu et al., 2025). These dimensions are particularly relevant in blended learning environments because digital tools and interactive classroom activities can support multiple forms of engagement simultaneously.

Another important theoretical perspective is constructivist learning theory, which suggests that students learn more effectively when they actively construct knowledge through interaction, reflection, and collaboration (Brown et al., 2005; Maric et al., 2025). Blended learning environments often support this process by combining online resources with face-to-face discussions, allowing students to explore ideas, exchange perspectives, and apply concepts in practical contexts. In addition, the Community of Inquiry (CoI) framework proposed by Joksimovic et al. (2014) highlights the importance of three elements in online and blended learning environments: cognitive presence, social presence, and teaching presence. Cognitive presence refers to the development of understanding through reflection and discourse, social presence relates to interaction and collaboration among students, and teaching presence involves the design and facilitation of learning experiences by instructors (Garrison and Akyol, 2013; Kovanović et al., 2015). Together, these theoretical perspectives provide a useful foundation for understanding how blended learning may influence student engagement in higher education. With Nepal focusing more on digital learning, it is important to study the benefits and challenges of blended learning for student engagement in these programs.

In South Asia, countries like India (UGC, 2020), Pakistan (Rasheed et al., 2020) and Sri Lanka (Perera et al., 2021) have promoted blended learning in higher education. In India, programs like SWAYAM provide online learning platforms that are included in university courses. In Pakistan, the Higher Education Commission has encouraged colleges to use blended learning, especially during and after the COVID-19 pandemic, although issues like infrastructure and teacher training remain challenges. In Sri Lanka, blended learning has been introduced in teacher training and other higher education programs to improve access and quality. In Nepal, the Ministry of Education has promoted digital teaching and student-centered learning (Bastola et al., 2024; Rijal et al., 2024), but progress has been uneven because of differences in internet access, institutional resources and teacher readiness (Joji et al., 2022; Balalle, 2024). With more digital technology available and growing demand for flexible learning, blended learning is likely to become more popular in Nepal, particularly in undergraduate business programs where teamwork, analytical skills and problem solving are important (Khadka and Acharya, 2023).

Because blended learning is becoming more common in South Asia, this study looks at how Nepali undergraduate business students experience it, especially in terms of their engagement in class activities. Over the past decade, Nepal’s higher education policies have focused on student-centered teaching and the use of digital technology to improve learning outcomes (Khadka et al., 2025; Shrestha et al., 2022). Research shows that well-designed blended learning can increase motivation, participation and overall learning (Ghimire et al., 2022; Laudari et al., 2021). However, despite these policies and programs, we still know little about how Nepali students actually engage in blended classrooms and whether these methods meet policy goals.

Building on these theoretical perspectives and the limited empirical research in the Nepali context, this study examines how blended learning influences student engagement among undergraduate business students. This study therefore aims to answer two questions:

- How do undergraduate business students in Nepal experience engagement in blended learning?
- What opportunities and challenges do blended learning approaches bring for student engagement in Nepali higher education?

The results are expected to help understand how effective blended learning is in Nepal and provide suggestions to improve teaching and student engagement in undergraduate business programs.

## 2 Literature review

### 2.1 Blended learning and student engagement

The main goal of blended learning in higher education is to improve teaching and learning by combining face-to-face classes with online learning tools. This approach asks teachers to use methods that not only deliver content but also actively involve students in learning. In general, blended learning can follow two teaching styles: traditional teacher-centered and modern student-centered. The traditional style focuses on lectures, memorization and one-way teaching, where students mainly receive information (Ahmed et al., 2022a; Rennie et al., 2007). The modern student-centered style, based on constructivist ideas, treats students as active learners who participate in discussions, group work and activities that use technology (Khadka et al., 2024; Khadka et al., 2022).

In traditional education, lectures have been the main teaching method, especially in South Asia, where teachers are seen as the main authority in the classroom (Dahal and Manandhar, 2023). But with the growth of digital tools, blended learning has brought new ways of teaching. These include: (1) online discussion forums and group tasks that let students share ideas and think critically (Thapaliya et al., 2024); (2) problem-based learning, where students apply what they learn to real business situations; (3) role-playing and simulations using online platforms to improve critical thinking and decision-making skills (Poudel et al., 2020); and (4) group activities that mix classroom and online work to build teamwork and communication skills (Dangal, 2025). Using technology in this way has helped move teaching from being teacher-centered to more student-centered, with students taking a more active role in learning.

Technology plays an important role in blended learning by making learning more interactive, flexible and independent. *For example,* in India, online platforms like SWAYAM allow students to access a variety of learning materials and study on their own (UGC, 2020). In Pakistan and Sri Lanka, using digital tools in blended classrooms has helped improve student participation and learning outcomes, although problems with infrastructure and digital skills still exist (Adhikari, 2021; Timsina and Pokhrel, 2026). In Nepal, the Ministry of Education, Science and Technology has focused on using digital teaching methods to improve education quality and the COVID-19 pandemic has sped up the use of online platforms (Iraki, 2025). Recent research shows that when blended learning is well designed, it can increase students’ motivation, active participation and overall engagement (Baral et al., 2024).

However, blended learning also faces several challenges. In Nepal, problems such as poor internet access, limited support from institutions and insufficient teacher training make it difficult to implement effectively (Gyawali et al., 2024; Yadav et al., 2018). Students from rural areas may have trouble accessing reliable digital tools, which can lead to uneven participation and engagement. These challenges can affect learning outcomes, especially in skill-based programs like undergraduate business studies, where teamwork, problem solving and critical thinking are important. This aligns with global studies that emphasize the need to combine digital tools, teaching methods and institutional support to ensure students are truly engaged in blended learning (Pokhrel et al., 2025; Upadhayaya et al., 2021).

So, even though blended learning has great potential to improve student engagement, especially in undergraduate business programs, its effectiveness in Nepal is still not well studied. Previous research highlights the need for studies focused on local contexts, examining not just how blended learning is used, but also how students experience and respond to it. Understanding this will give better insight into the opportunities and challenges of using blended learning to engage students in Nepal’s higher education.

Student engagement is widely recognized as a central factor influencing learning outcomes in higher education. Researchers commonly describe engagement as a multidimensional concept that includes students’ active involvement in academic activities, their emotional connection to learning, and their investment in understanding course content. According to Fazel and Sayaf (2025), student engagement can be understood through three main dimensions: behavioral, cognitive, and emotional engagement. Behavioral engagement refers to observable participation in academic activities such as attending classes, completing assignments, and participating in discussions. Cognitive engagement involves students’ willingness to invest effort in understanding complex ideas, solving problems, and applying knowledge critically. Emotional engagement relates to students’ interest, motivation, and positive feelings toward learning activities and the learning environment.

These dimensions provide a useful framework for understanding how students interact with learning environments, including blended learning contexts. In blended learning environments, behavioral engagement may appear through participation in online discussions, collaborative tasks, or classroom debates. Cognitive engagement may occur when students analyze case studies, solve business problems, or apply theoretical knowledge to practical situations. Emotional engagement can develop when students feel motivated, interested, or confident while interacting with digital tools and collaborative activities.

Previous research shows that learning environments that combine active learning strategies with digital tools can strengthen all three dimensions of engagement. For example, Sarchet et al. (2025) explains that student engagement develops when learning activities encourage interaction, reflection, and meaningful participation. Similarly, Horsley et al. (2025) highlight that blended learning environments often promote higher levels of engagement because students have multiple opportunities to interact with content, teachers, and peers both inside and outside the classroom. Including these psychological dimensions helps explain why blended learning can influence not only students’ academic performance but also their motivation, participation, and attitudes toward learning.

### 2.2 Psychological dimensions of student engagement

Student engagement is increasingly viewed as a psychological process that reflects how students think, feel, and behave during learning activities. The three dimensions of engagement behavioral, cognitive, and emotional are closely connected and together influence academic success. Behavioral engagement focuses on students’ participation in academic activities such as attending classes, completing assignments, and contributing to group discussions. In blended learning environments, this may include participating in online forums, completing digital quizzes, and collaborating on group projects.

Cognitive engagement refers to the mental effort students invest in learning tasks. Students who are cognitively engaged attempt to understand ideas deeply, analyze problems, and apply knowledge to new situations. Blended learning environments often support cognitive engagement by providing opportunities for problem-based learning, case analysis, and reflective discussion. For example, students may review online materials before class and then apply their understanding during in-person discussions or collaborative activities.

Emotional engagement reflects students’ attitudes, interest, and motivation toward learning. When students feel interested, confident, and supported in their learning environment, they are more likely to remain engaged and persist in challenging tasks. Research shows that interactive learning activities and collaborative environments can improve emotional engagement by making learning more enjoyable and meaningful (Fortune, 2025).

Understanding these psychological dimensions is important for evaluating the effectiveness of blended learning environments. When instructional design supports behavioral participation, cognitive effort, and emotional motivation simultaneously, students are more likely to achieve deeper learning outcomes and develop important academic skills.

### 2.3 Active learning and its application in blended learning contexts

Active learning strategies are closely connected to the concept of student engagement because they encourage students to participate actively in the learning process. Rather than focusing only on lectures and information delivery, active learning requires students to interact with content, solve problems, discuss ideas with peers, and reflect on their understanding. When active learning strategies are integrated into blended learning environments, they can strengthen behavioral, cognitive, and emotional engagement simultaneously. For example, group discussions encourage behavioral participation, case analysis supports cognitive engagement, and collaborative activities can increase motivation and emotional involvement in learning.

Active learning in higher education focuses on students being actively involved in learning through discussions, reflections, problem solving and working with teachers and classmates. Unlike traditional teaching, where students mainly listen to lectures, active learning asks students to engage directly with ideas, tasks and real-world problems. This student-centered approach emphasizes *“learning by doing,”* because activities like discussions, role-plays and hands-on exercises help students understand and use knowledge better. In blended learning, active learning is even more important, as it combines classroom and online activities, giving students more chances to interact with content and each other.

Research in South Asia and other regions shows that when blended learning uses active learning strategies, students become more motivated, think more deeply and work better in teams. Activities like case studies, online discussions with peers, group projects and simulations help students engage and develop critical thinking skills in undergraduate programs. *For example,* online discussion forums and collaborative problem-solving tasks through learning management systems (LMS) let students interact and learn outside the classroom. Similarly, flipped classrooms, where students watch digital content before class and then do active learning tasks during in-person sessions, have been found to improve participation and performance.

Active learning in blended learning can be done with or without technology. Without technology, students can take part in activities like group debates, case studies and peer teaching in class. With technology, tools like gamified platforms (e.g., Kahoot!), interactive mobile apps and video-based problem-solving tasks can help increase student engagement. In higher education, learning management systems (LMS) also let teachers add quizzes, polls and reflective journals, keeping students engaged continuously.

Even though blended learning offers many benefits, there are still challenges. Many teachers and students have limited digital skills, internet access can be unreliable, and schools may not provide enough support. These problems make it harder for students to take part in group activities and reduce the effectiveness of technology-based learning. Also, if teachers are not properly trained, they may continue to focus mostly on lectures, which limits students’ chances to think critically and participate actively.

The review above shows that active learning is becoming more important in blended learning research around the world, as it helps improve student engagement and learning. However, in Nepal, we do not yet know much about how undergraduate business programs are using active learning in blended classrooms. This study helps fill that gap by exploring how Nepali undergraduate students experience active learning in blended learning and by identifying the challenges and opportunities in using these strategies.

## 3 Methods

### 3.1 Research design

This study employed a sequential mixed-methods explanatory design, combining quantitative and qualitative approaches to explore undergraduate business students’ engagement in blended learning in Nepal. The design involved two phases: first, quantitative data were collected to measure students’ knowledge, engagement, and motivation; second, qualitative data were gathered to understand students’ experiences and perceptions of blended learning in depth (Ivankova et al., 2006). This approach allows the numerical data to be contextualized and explained through rich descriptions of student experiences.

The quantitative phase included pre- and post-tests designed to assess learning outcomes, engagement, and collaborative skills, supplemented by structured surveys to measure students’ engagement and motivation. The surveys were a distinct instrument from the pre- and post-tests, developed to capture students’ self-reported experiences of participation, interest, and enthusiasm in blended learning activities. The qualitative phase employed a phenomenological approach, which emphasizes understanding individuals’ lived experiences as they perceive and interpret them (van Manen, 2017). This approach was chosen because the study sought to explore how students interpret their engagement in blended learning rather than merely quantify outcomes.

### 3.2 Context and participants

The study was conducted at three colleges in Nepal offering undergraduate business programs. Two of these institutions were public colleges, and one was private. These colleges were selected to capture a diversity of teaching approaches, facilities, and access to technology. The study was conducted over 6 weeks during a single academic semester.

In the quantitative phase, 120 undergraduate business students participated. Students were recruited using a stratified random sampling procedure to ensure representation from all 4 years of the undergraduate business program. First, enrollment lists were obtained from the academic offices of the three participating colleges. Students were then grouped into four strata based on their year of study (first year, second year, third year, and fourth year). From each stratum, participants were selected using a random number generator. The number of students selected from each year level was proportional to the size of the cohort in each institution. This approach ensured that students with different levels of academic experience and exposure to digital learning were included in the sample.

A total of 120 students participated in the quantitative phase. Among them, 48 students (40%) were male and 72 students (60%) were female, with ages ranging from 18 to 24 years. The distribution across year levels was relatively balanced, which helped capture variation in students’ learning experiences and technological familiarity. For the qualitative phase, 12 students volunteered to participate in focus group interviews. Convenience sampling was used at this stage because participation depended on students’ availability after the intervention. Efforts were made to ensure diversity among participants, including representation from both public and private colleges as well as different year levels and gender groups.

### 3.3 Research procedures and intervention

The study implemented a six-week blended learning intervention that combined face-to-face instruction with structured online learning activities. The instructional design followed an active learning framework, where students engaged in collaborative tasks, problem solving activities, and digital learning interactions both inside and outside the classroom. Each week included two in-person sessions of approximately 90 min and two online sessions lasting about 60 min. The online components were delivered through the institution’s Learning Management System (LMS), which provided access to course materials, discussion forums, quizzes, and collaborative tools. In addition, students used widely available digital tools such as Google Docs for group assignments and Kahoot! for gamified quizzes designed to reinforce learning. The intervention followed a flipped classroom approach. Before attending in-person sessions, students accessed digital learning materials through the LMS, including recorded lectures, short readings, and instructional videos. During classroom sessions, students participated in interactive activities such as case study analysis, group discussions, role-playing simulations, and collaborative problem-solving exercises related to business scenarios.

Online discussion forums were organized weekly, where students responded to case-based questions and provided feedback on their peers’ responses. These discussions encouraged critical reflection and allowed students to engage with course topics beyond classroom time. Gamified quizzes were used at the end of each module to reinforce key concepts and provide immediate feedback on learning progress. Instructors played an active facilitation role throughout the intervention. During face-to-face sessions, instructors guided discussions, clarified complex concepts, and monitored collaborative group work. In the online environment, instructors moderated discussion forums, provided feedback on assignments, and addressed students’ questions through the LMS messaging system. This combination of structured online learning and instructor-supported classroom interaction was designed to promote active engagement and deeper understanding of course content.

### 3.4 Instrumentation and measures

#### 3.4.1 Pre- and post-tests

The pre- and post-tests consisted of 20 multiple-choice questions designed to measure three domains: (1) conceptual understanding of blended learning and business course content, (2) awareness of teamwork and collaborative learning practices, and (3) engagement and motivation related to active learning activities. Each question had four response options with one correct answer. The maximum possible score was 20. To ensure content validity, the instrument was reviewed by two experts in business education and instructional design, each with more than 10 years of teaching experience in higher education. The experts evaluated the clarity, relevance, and alignment of each question with the study objectives and suggested minor revisions to wording and structure. A pilot test was conducted with 15 undergraduate business students from a college that did not participate in the main study. The pilot test assessed the clarity of instructions, the appropriateness of question difficulty, and the overall reliability of the instrument. Based on the pilot results, several items were revised to improve clarity. The internal consistency reliability of the instrument was assessed using Cronbach’s alpha, which produced a value of 0.81, indicating acceptable reliability for educational research (Table 1). These validation procedures helped ensure that the test measured the intended constructs and produced consistent results.

**Table 1:** Sample pre- and post-test questions.

Question	Options
1. Which of the following best describes blended learning?	A. Only classroom teaching B. Only online lectures C. A mix of online and face-to-face teaching D. Self-study only
2. What is the main purpose of active learning?	A. Listening to lectures B. Memorizing definitions C. Engaging students through activities and problem solving D. Preparing for exams only
3. Which of the following is an example of asynchronous learning activity?	A. Online discussion forums B. Classroom role-play C. Teacher-guided debates D. In-person case study presentation

#### 3.4.2 Engagement and motivation survey

In addition to the pre- and post-tests, a structured engagement and motivation survey was administered at the same time points. The survey included Likert-scale items measuring interest in learning activities, participation frequency, perceived usefulness of online and in-person tasks, and willingness to collaborate with peers. The survey was a separate instrument designed specifically to capture subjective dimensions of engagement and motivation not directly assessed through knowledge-based test items.

#### 3.4.3 Focus group interviews

Focus group interviews were conducted after the intervention to explore students’ experiences and perceptions of blended learning. Each session lasted approximately 90 min and was conducted via Zoom or Microsoft Teams to accommodate participants from multiple colleges. Interviews were semi-structured, with 13 guiding questions that addressed participation in blended learning activities, challenges with technology, teamwork, critical thinking, and overall engagement (Table 2). Sessions were audio-recorded with consent and transcribed for analysis.

**Table 2:** Focus group interview questions.

Question
1. How do you usually take part in blended learning classes?
2. Which online activities (forums, quizzes, videos) do you enjoy most?
3. What problems have you faced in using online resources?
4. How do classroom discussions and group projects affect your engagement?
5. Do you feel more motivated in blended classes compared to traditional classes? Why or why not?
6. How does blended learning affect your teamwork and collaboration skills?
7. What role does technology play in your participation?
8. What difficulties do you face with digital assignments?
9. How does internet connectivity affect your participation?
10. What are the main differences between your online and in-person engagement?
11. How does blended learning help with problem solving and critical thinking?
12. What improvements would you suggest for blended learning in your program?
13. How has your overall engagement changed after taking part in blended learning?

### 3.5 Data analysis

Quantitative data from pre- and post-tests and surveys were analyzed to examine changes in knowledge, engagement, and collaboration. Descriptive statistics, including means, standard deviations, and percentiles, were calculated for all measures (Table 3). To assess the effect of the intervention, the Wilcoxon signed-rank test was used instead of a paired-samples t-test because initial analysis of the pre-test scores indicated non-normal distributions and the presence of outliers. Non-parametric testing allowed for comparison of paired ordinal and interval-level data without assuming normality. A significance level of *p* < 0.05 was applied. In addition to statistical significance, an effect size was calculated to determine the magnitude of the observed change between pre-test and post-test scores. For the Wilcoxon signed-rank test, the effect size was calculated using the formula $r=Z/\sqrt{N}$ , where *Z* represents the test statistic and *N* represents the total number of observations (Yang et al., 2022). Reporting the effect size provides a clearer understanding of the practical importance of the intervention beyond statistical significance. Qualitative data from focus groups were analyzed using thematic analysis to explore students’ lived experiences and perspectives (Basnet et al., 2024; Marahatta and Dixit, 2008). This process involved six phases:

**Table 3:** Phases of thematic analysis.

Phase	Description
1. Getting to know the data	Transcribing, reading and re-reading transcripts, noting initial thoughts
2. Generating initial codes	Systematically coding significant features across the dataset
3. Searching for themes	Collating codes into potential themes and sub-themes
4. Reviewing themes	Comparing themes against coded extracts and the overall dataset for coherence
5. Defining and naming themes	Refining each theme, naming them clearly to reflect content
6. Producing the report	Selecting illustrative extracts, analyzing them, and linking with literature

Focus group participants were anonymized using labels (S1, S2, etc.) to protect confidentiality. The qualitative data were then used to contextualize and explain the quantitative results, providing a comprehensive understanding of student engagement in blended learning.

This methodological framework ensured that both measurable changes in knowledge and subjective experiences were systematically captured. The combination of pre- and post-tests, structured surveys, and focus group interviews provided complementary data, allowing for triangulation and enhanced validity of findings. To strengthen the credibility of the qualitative findings, the thematic analysis process included peer review of coding decisions and repeated comparison between themes and original transcripts. Selected quotations were used to illustrate each theme and ensure that interpretations accurately reflected participants’ experiences.

### 3.6 Ethics statement

Ethical approval for this study was obtained from the Ethics Review Committee of the Divya Jyoti Community Organization, Hetauda, Nepal, which served as the institutional ethics body overseeing the research. All participants in the study were undergraduate students aged 18 years or above and participated voluntarily. Prior to data collection, the purpose of the study, procedures, potential risks, and benefits were clearly explained to participants in the local language. Because some participants had limited literacy, verbal informed consent was obtained instead of written consent, following the recommendation and approval of the ethics committee. No parental or guardian consent was required because all participants were adults. Participants were informed that they could decline to answer any question or withdraw from the study at any time without any consequences. Confidentiality and anonymity were strictly maintained, and no personally identifiable information was recorded. All data were used solely for academic research purposes.

## 4 Results

### 4.1 Effects of blended learning on knowledge, engagement and collaboration

The quantitative phase of this study aimed to measure changes in Nepali undergraduate business students’ knowledge, engagement, and collaborative skills following a six-week blended learning intervention. A total of 120 students participated in pre- and post-tests, as well as in a structured engagement and motivation survey. The pre- and post-tests consisted of 20 multiple-choice questions (MCQs), which assessed knowledge acquisition, awareness of teamwork, and aspects of engagement and motivation.

Although MCQs are typically considered suitable for assessing cognitive outcomes, the items in this study were specifically designed to measure affective and behavioral aspects of engagement and motivation in addition to knowledge. For example, items probed students’ frequency of participation in collaborative tasks, interest in active learning, willingness to contribute to group discussions, and perceived value of technology-supported learning activities. The content and clarity of these items were validated by two business education experts, and a pilot test with 15 students ensured that the questions were comprehensible and capable of capturing subjective experiences reliably. The survey items used a Likert-type scale to quantify engagement and motivation, while the MCQs captured applied knowledge and awareness of teamwork strategies. Together, these instruments provided a comprehensive assessment of students’ cognitive, affective, and behavioral engagement in blended learning.

Descriptive statistics, including mean scores, standard deviations, and percentiles, were calculated for both pre-test and post-test results to provide a clear picture of students’ performance before and after the intervention. These statistics were complemented with inferential analysis using the Wilcoxon signed-rank test, a non-parametric method suitable for comparing paired scores when data distributions are non-normal or contain outliers. The choice of this test ensured that changes in knowledge, engagement, and collaborative skills were assessed rigorously without violating statistical assumptions.

### 4.2 Pre-test and post-test performance

The pre-test results showed that students had a moderate level of prior knowledge and awareness regarding blended learning, teamwork, and active participation. The mean pre-test score was 11.32 out of 20 (SD = 3.45), with a median of 11.00, indicating a balanced distribution of performance across the sample. The 25th and 75th percentiles were 9.00 and 14.00, respectively, suggesting some variation in students’ initial understanding and engagement levels. Table 4 presents a detailed summary of the descriptive statistics for pre- and post-test scores.

**Table 4:** Descriptive statistics of pre- and post-test scores.

Test	*N*	Mean	Std. deviation	Minimum	Maximum	25th percentile	Median	75th percentile
Pre-test	120	11.32	3.451	4	20	9.00	11.00	14.00
Post-test	120	16.45	2.987	9	20	15.00	17.00	19.00

After six weeks of blended learning, students’ post-test scores increased substantially. The mean post-test score rose to 16.45 (SD = 2.99), with a median of 17.00, reflecting consistent improvement in both knowledge and engagement. The lower standard deviation compared to the pre-test indicates that students’ understanding and participation became more uniform following the intervention. The increase from 11.32 to 16.45 represents a substantial improvement in both cognitive understanding and affective engagement, demonstrating that the structured use of blended learning and active learning strategies had a positive impact.

**Figure 1:**
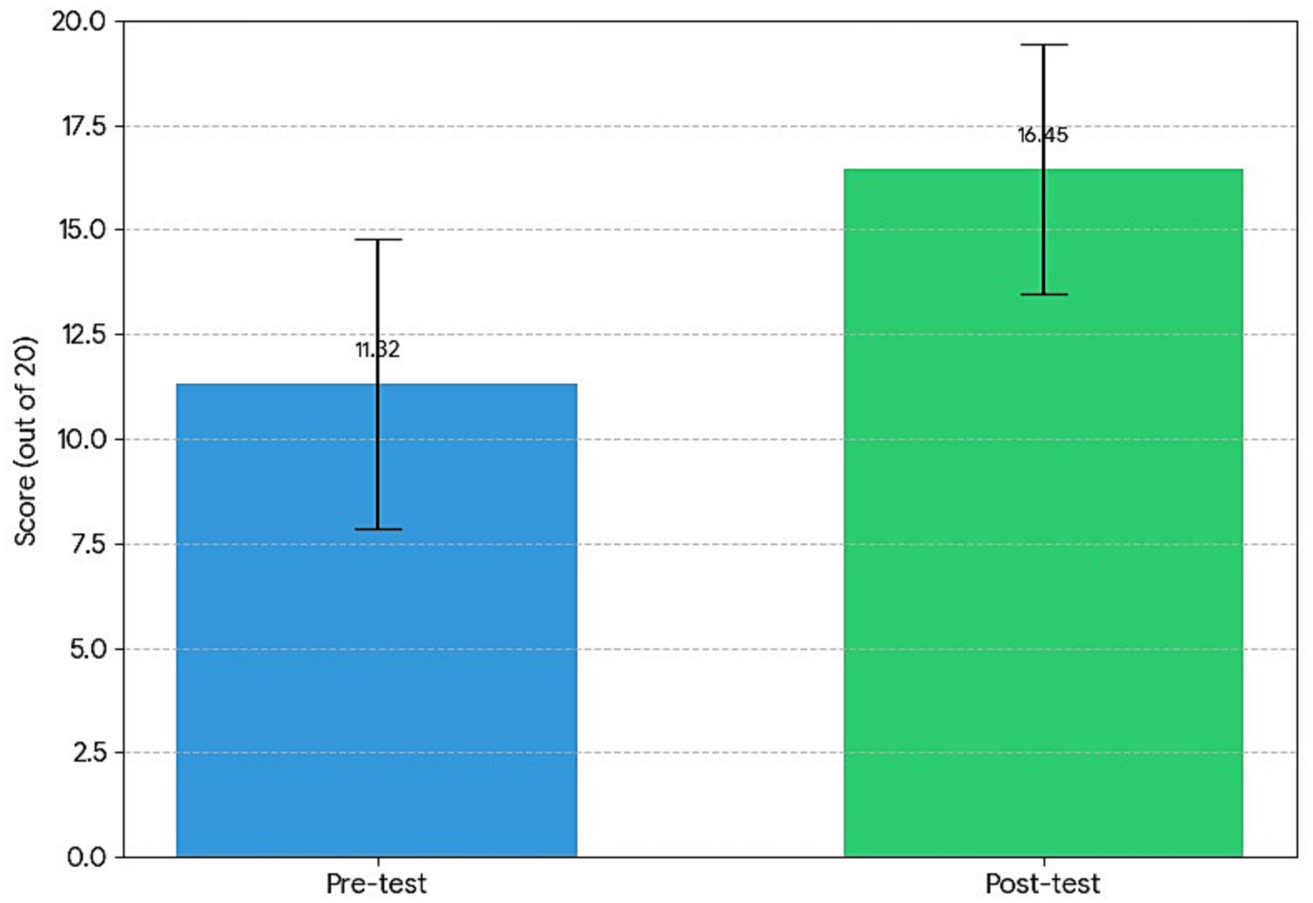
Comparison of mean pre-test and post-test scores.

To enhance clarity for readers, Figure 1 provides a visual comparison of mean pre- and post-test scores. This bar chart demonstrates the magnitude of improvement clearly and highlights the effectiveness of the intervention in increasing students’ knowledge and engagement.

### 4.3 Inferential analysis: Wilcoxon signed-rank test

To determine whether the observed improvements were statistically significant, the Wilcoxon signed-rank test was performed. This non-parametric test was chosen instead of a paired-samples t-test because the pre-test scores exhibited non-normal distributions and some outliers (Table 5). The Wilcoxon test compares paired observations and is well-suited for ordinal or interval-level data where parametric assumptions cannot be guaranteed.

**Table 5:** Pre- and post-test comparison using Wilcoxon signed-rank test.

Ranks	*N*	Mean rank	Sum of ranks
Negative ranks a	2	5.50	11.00
Positive ranks b	118	60.23	7107.00
Ties c	0		
Total	120		

a, post-test < pre-test; b, post-test > pre-test; c, post-test = pre-test.

Results showed that 118 students had higher post-test scores than their pre-test scores, while only two students scored lower, and no cases were tied. The Wilcoxon signed-rank test yielded a Z-value of −5.127 with a *p*-value <0.001, indicating a statistically significant increase in performance across the sample (Table 6). To better understand the magnitude of this improvement, the effect size was calculated using the formula $r=Z/\sqrt{N}$ . Based on the test statistic (Z = −5.127) and the sample size (*N* = 120), the effect size was *r* = 0.47, which represents a moderate to large effect according to common interpretation guidelines. This result indicates that the blended learning intervention had a meaningful impact on students’ knowledge, engagement, and collaboration outcomes, beyond statistical significance alone. These results confirm that the blended learning intervention effectively enhanced both knowledge and engagement among the participants.

**Table 6:** Wilcoxon signed-rank test results.

Test	*Z*	Asymp. Sig. (two-tailed)
Post-test–pre-test	−5.127	0.000

These results strongly support the conclusion that students benefited from the blended learning intervention. The combination of structured online and in-person activities, guided by active learning principles, resulted in significant gains in knowledge, collaborative skills, and self-reported engagement.

### 4.4 Engagement and motivation survey results

To complement the MCQ-based assessment, a structured engagement and motivation survey captured students’ self-reported interest, participation, and enthusiasm in blended learning activities. The survey included 15 Likert-scale items ranging from 1 (“strongly disagree”) to 5 (“strongly agree”), assessing four domains: (i) interest in online and in-person activities, (ii) participation frequency, (iii) perceived usefulness of tasks, and (iv) willingness to collaborate.

Survey analysis indicated that students’ engagement improved across all domains. The mean score for interest in blended activities increased from 3.12 (pre-intervention) to 4.25 (post-intervention), suggesting that students became more motivated and curious about course activities. Participation frequency scores rose from 2.98 to 4.10, while perceived usefulness of activities improved from 3.05 to 4.18. Collaboration willingness increased from 3.20 to 4.22 (Table 7). These changes were statistically significant, as confirmed by paired Wilcoxon signed-rank tests for each domain (all *p* < 0.001).

**Table 7:** Pre- and post-intervention engagement and motivation survey scores with effect sizes.

Domain	Pre-test mean	Post-test mean	Change	*p*	Effect size (*r*)
Interest in activities	3.12	4.25	+1.13	<0.001	0.65
Participation frequency	2.98	4.10	+1.12	<0.001	0.63
Perceived usefulness	3.05	4.18	+1.13	<0.001	0.64
Willingness to collaborate	3.20	4.22	+1.02	<0.001	0.60

Effect size *r* is calculated as *Z*/√*N* for Wilcoxon signed-rank tests. Values above 0.5 are considered large effects, suggesting the intervention had a strong impact on engagement and motivation.

**Figure 2:**
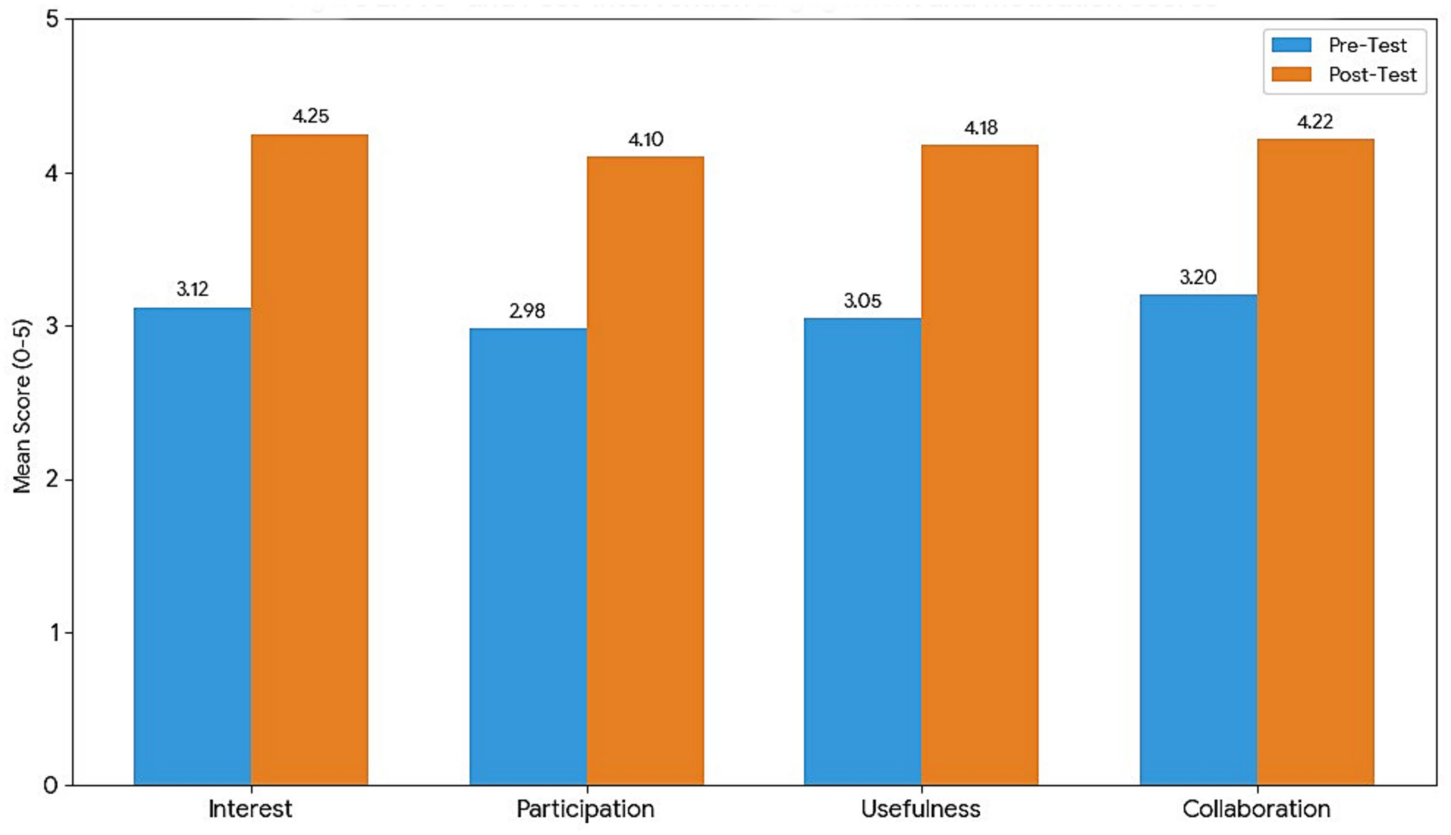
Pre- and post-intervention engagement and motivation scores.

The survey results align closely with the MCQ findings, indicating that knowledge gains were accompanied by higher engagement, motivation, and collaboration readiness. Figure 2 provides a visual summary of the change in engagement and motivation across these four domains.

These data indicate that the blended learning approach not only improved cognitive outcomes but also supported students’ affective engagement, reinforcing the importance of combining active learning strategies with technology-enhanced instruction.

### 4.5 Students’ experiences of engagement in blended learning

The qualitative findings provide deeper insight into how undergraduate business students in Nepal experienced engagement in blended learning environments. The focus group interviews helped explain the patterns observed in the quantitative data and provided detailed descriptions of how students interacted with both online and face-to-face learning activities. Twelve students from three colleges participated in two focus group discussions conducted after the six-week blended learning intervention.

Thematic analysis of the interview transcripts identified several key themes that reflect students’ experiences of engagement. These themes include active participation in blended learning activities, development of critical thinking and teamwork skills, the role of digital tools in learning, motivational aspects of blended learning, and the challenges encountered during participation. The qualitative findings also help clarify how engagement was experienced by students beyond the measurement captured in the quantitative phase. In particular, the interview data reveal how students interpreted engagement through participation, interaction, interest, and collaboration within the blended learning environment.

#### 4.5.1 Understanding engagement in the blended learning context

Students described engagement in blended learning as a combination of participation in classroom discussions, involvement in online tasks, and collaboration with peers. Rather than viewing engagement simply as attending lectures or completing assignments, many students associated engagement with active involvement in learning activities.

For example, one participant explained that engagement increased when learning involved interaction rather than passive listening. As one student noted:

“In blended classes we are not just listening to the teacher. We discuss topics, solve cases and share ideas online. This makes us feel more involved in learning.” (S4)

Another student described how engagement occurred through the process of applying theoretical knowledge to practical problems.

“When we analyze business cases together and discuss them online later, I feel that I understand the topic better. It is more interesting than just reading the book.” (S2)

These comments suggest that students perceived engagement as an active learning process rather than simple participation. This observation aligns with the constructivist learning perspective discussed earlier in the literature review, where students build knowledge through interaction, collaboration, and reflection.

#### 4.5.2 Participation in active learning activities

One of the strongest themes that emerged from the interviews was the importance of active learning activities in promoting engagement. Students consistently reported that activities such as case studies, group discussions, online forums, and gamified quizzes helped them remain interested and involved in the course.

Many students emphasized that these activities encouraged them to think about business problems more carefully. For example, one student explained:

“In the online discussion forums we have to explain our opinions and respond to other students. This makes us think more about the topic before we write.” (S6)

Another participant described how group projects created opportunities for collaborative learning.

“Working in groups helps us share ideas and understand different viewpoints. Sometimes my classmates explain concepts in a way that is easier to understand.” (S9)

Students also highlighted the value of case-based learning in bridging the gap between theory and practice. Through case studies, students were able to analyze real-world business situations and apply classroom knowledge.

“When we study real business cases, we can see how the theory works in practice. This makes the lesson more meaningful.” (S3)

These findings suggest that active learning strategies play an important role in promoting engagement within blended learning environments. Rather than focusing solely on knowledge transmission, these activities encourage students to interact with content, peers, and instructors.

#### 4.5.3 Development of critical thinking and problem-solving skills

Another important theme emerging from the qualitative data is the development of higher-order thinking skills. Many students reported that blended learning activities encouraged them to analyze information, evaluate alternatives, and propose solutions to business problems.

One student described how group discussions supported critical thinking.

“When we discuss different business strategies, we compare ideas and try to find the best solution. This helps us think more deeply about the topic.” (S1)

Another student explained that online forums provided time for reflection before responding.

“In online discussions we can read other comments first and then write our response. This gives us time to think carefully.” (S7)

These experiences reflect the emphasis on critical thinking highlighted in the literature on active learning. According to students, the blended learning approach created opportunities to practice analytical thinking both inside and outside the classroom.

Students also noted that collaborative problem-solving tasks improved their ability to work in teams.

“Group projects require cooperation. We divide tasks, discuss ideas and combine our work. It improves our teamwork skills.” (S10)

This theme demonstrates that engagement in blended learning is closely connected to the development of academic and professional skills. Through interactive activities, students were able to practice communication, problem solving, and decision-making skills relevant to business education.

#### 4.5.4 Role of digital tools in supporting engagement

Students also discussed the role of digital tools in facilitating their participation in blended learning. The Learning Management System (LMS) allowed them to access course materials, participate in discussion forums, submit assignments, and complete quizzes.

Many students appreciated the flexibility offered by digital platforms. One participant commented:

“The online system allows us to review lectures and materials anytime. If we miss something in class, we can check it again online.” (S8)

Another student highlighted the convenience of online collaboration tools.

“We use Google Docs and online forums to work together on assignments. It is easier to share ideas and edit our work.” (S11)

Students also noted that digital quizzes made learning more interactive and enjoyable. Gamified quizzes provided immediate feedback and helped reinforce learning.

“The quizzes are interesting because we can see our results immediately. It also motivates us to study more.” (S5)

These comments suggest that technology can support student engagement when it is integrated with meaningful learning activities. Digital tools allowed students to interact with content and collaborate with peers beyond the physical classroom.

#### 4.5.5 Motivation and interest in learning

Students frequently reported that blended learning increased their motivation and interest in studying business subjects. Many participants explained that the variety of learning activities made classes more engaging compared to traditional lecture-based instruction.

One student described the difference clearly:

“In traditional classes we mostly listen to lectures, but in blended classes we participate more. This makes the class more interesting.” (S2)

Another student emphasized the role of interactive activities in maintaining motivation.

“When we have discussions, quizzes and group work, we feel more active. It keeps us motivated to learn.” (S6)

Several participants also mentioned that blended learning increased their confidence in expressing ideas.

“The online forums help shy students participate because we can write our opinions even if we are not comfortable speaking in class.” (S12)

These observations demonstrate that engagement is not limited to cognitive participation but also includes emotional and motivational aspects. Students felt more interested and confident when learning involved interactive and collaborative tasks.

#### 4.5.6 Challenges in participating in blended learning

Despite the positive experiences reported by many students, several challenges were also identified. The most common difficulty was unreliable internet connectivity. Students explained that poor internet access sometimes prevented them from participating fully in online activities.

One participant stated:

“Sometimes the internet connection is slow, and it is difficult to join online discussions or upload assignments.” (S4)

Another challenge was limited familiarity with digital platforms. Some students reported that they needed time to learn how to use the LMS and other online tools.

“At first it was difficult to use the system because we were not familiar with it. But after some practice it became easier.” (S9)

Students also mentioned difficulties in balancing online tasks with in-person coursework.

“Managing both online assignments and classroom activities can be challenging if there are many deadlines.” (S1)

These challenges highlight the importance of institutional support and digital training for successful implementation of blended learning. Students suggested that introductory training sessions on digital tools could help improve participation and reduce technical difficulties.

## 5 Discussion

This study examined how blended learning influences knowledge, engagement, and collaboration among undergraduate business students in Nepal. The results show that a structured blended learning approach combining online and face-to-face activities significantly improved students’ understanding of business concepts, participation in learning activities, and collaboration with peers. Both the quantitative and qualitative findings provide complementary evidence that blended learning can enhance student engagement when active learning strategies and digital tools are effectively integrated.

The quantitative results demonstrated a clear improvement in students’ performance after the six-week intervention. The mean post-test score increased from 11.32 to 16.45, and the Wilcoxon signed-rank test confirmed that this improvement was statistically significant. This result suggests that blended learning activities such as online discussions, case studies, collaborative tasks, and gamified quizzes can strengthen both knowledge acquisition and engagement. These findings are consistent with earlier research indicating that blended learning environments promote deeper learning and increased participation. For example, Holbrey (2020) found that students in blended learning environments often perform better than those in traditional lecture-based settings because they have more opportunities to interact with learning materials and apply knowledge through practical tasks.

The engagement and motivation survey also showed substantial improvement in students’ interest, participation, perceived usefulness of activities, and willingness to collaborate. These findings support previous studies suggesting that blended learning increases motivation and participation by combining flexible online learning with interactive classroom experiences. Pham Thi et al. (2025) reported that blended learning allows students to engage with course content through multiple channels, including digital platforms, discussions, and collaborative tasks, which can increase both cognitive and emotional engagement.

The qualitative findings further explain these quantitative improvements by describing how students experienced the blended learning environment. Students reported that activities such as case-based discussions, online forums, and group projects helped them better understand business concepts and encouraged them to think critically about real-world problems. These results align with the constructivist learning perspective, which emphasizes that students learn more effectively when they actively participate in constructing knowledge through interaction and reflection. Similar findings were reported by Albarrak et al. (2021), who found that interactive learning activities in blended environments improved students’ problem-solving skills and conceptual understanding.

Another important finding from this study is the role of collaborative learning in enhancing engagement. Students explained that group discussions and online collaboration tools allowed them to exchange ideas, learn from peers, and develop teamwork skills. These experiences support previous research highlighting the importance of collaboration in blended learning environments. For instance, Zhou (2025) observed that collaborative digital platforms encourage students to share perspectives and develop communication skills, which are essential for professional development in business education. The improvement in collaboration willingness found in the survey results is therefore supported by students’ descriptions of teamwork activities during focus group discussions.

Digital tools also played a significant role in supporting engagement and flexibility in the learning process. Students appreciated the ability to access learning materials online, participate in discussions outside the classroom, and review content when needed. This flexibility is consistent with findings reported by Latchem and Jung (2009), who emphasized that technology-supported learning environments allow students to learn at their own pace and revisit course materials for better understanding. Similarly, Khanal (2024) noted that digital platforms such as learning management systems can enhance engagement when they are integrated with interactive teaching strategies.

Despite these positive outcomes, the study also identified several challenges related to the implementation of blended learning. The most common issue reported by students was unreliable internet connectivity, which sometimes limited their ability to participate in online discussions or submit assignments. Similar challenges have been documented in previous research conducted in developing countries. For example, Khadka et al. (2025) reported that limited digital infrastructure and inconsistent internet access remain major barriers to the effective implementation of blended learning in Nepal. In addition, some students in this study indicated that they initially struggled with unfamiliar digital tools, which affected their confidence and participation in online activities.

The findings also highlight the importance of digital literacy and institutional support in ensuring successful blended learning implementation. Students suggested that training sessions on using learning management systems and digital tools could improve participation and reduce technical difficulties. This recommendation aligns with research by Laubscher and Bosch (2025), which emphasized that both students and teachers need adequate training and support to fully benefit from blended learning environments.

This study has several limitations that should be considered when interpreting the findings. First, the research was conducted with a relatively small sample of undergraduate business students from only three colleges in Nepal. As a result, the findings may not be fully generalizable to other disciplines or educational contexts. Second, the study relied primarily on self-reported survey data and focus group discussions to measure engagement, which may introduce response bias. Classroom observations or learning analytics data could provide additional evidence of students’ engagement in blended learning activities. Third, the intervention lasted only 6 weeks, which may not capture the long-term effects of blended learning on student motivation, academic performance, and skill development. Another limitation of this study is the absence of a control group using traditional teaching methods. Because all participating students experienced the blended learning intervention, it is difficult to attribute the observed improvements solely to the intervention itself.

Despite these limitations, the study offers several practical implications for higher education institutions in Nepal. First, colleges should invest in reliable digital infrastructure and internet access to ensure that all students can participate fully in online learning activities. Second, institutions should provide training programs for both students and teachers to improve digital literacy and effective use of learning management systems. Third, instructors should continue to incorporate active learning strategies such as case studies, collaborative projects, and online discussions into blended courses to encourage deeper engagement and critical thinking.

In conclusion, this study demonstrates that blended learning can significantly enhance student engagement, knowledge acquisition, and collaboration among undergraduate business students in Nepal. When supported by appropriate technology, training, and student-centered teaching methods, blended learning has strong potential to improve higher education outcomes and prepare students with the skills required for modern professional environments.

## 6 Conclusion and future research

This study highlights that blended learning can significantly enhance engagement, knowledge acquisition, and collaborative skills among undergraduate business students in Nepal. Structured activities such as online discussion forums, group projects, case-based problem solving, and gamified quizzes encouraged active participation, improved critical thinking, strengthened teamwork, and deepened understanding of business concepts. Students reported greater motivation, interest, and confidence in learning when actively involved in both online and in-person tasks. The integration of digital tools, when combined with active learning strategies, played a key role in fostering flexibility, interaction, and self-directed learning. At the same time, challenges such as unreliable internet access, unfamiliarity with digital platforms, and difficulty balancing online and face-to-face activities were identified. Addressing these obstacles through improved digital infrastructure, targeted training for students and teachers, and careful instructional design is essential for maximizing the benefits of blended learning.

Future research could expand the scope of inquiry in several ways. Studies should examine blended learning across different disciplines, institutions, and regions in Nepal to determine whether these findings are consistent in varied contexts. Longitudinal research could provide insights into the sustained impact of blended learning on engagement, skill development, and academic performance. Incorporating classroom observations, learning analytics, and teacher perspectives would offer a more comprehensive understanding of engagement patterns and pedagogical effectiveness. Finally, exploring the interplay between digital literacy, technological access, and student-centered teaching strategies could help develop evidence-based guidelines for implementing blended learning more effectively in Nepalese higher education.

## Data Availability

The original contributions presented in the study are included in the article/supplementary material, further inquiries can be directed to the corresponding author.
